# Inflammation as a mediator between neck adipose tissue and tumor aggressiveness in hypopharyngeal and laryngeal squamous cell carcinoma

**DOI:** 10.1186/s40644-025-00913-w

**Published:** 2025-07-29

**Authors:** Yu Jiang, Xiaodong Ji, Shanshan Gao, Xiaohuang Yang, Qing Li, Zhuo Yu, Xilong Yang, Zhuo Shen, Jie Shen, Shuang Xia

**Affiliations:** 1 Department of Radiology, First Central Hospital of Tianjin Medical University, Nankai District, Tianjin, China; 2 Department of Radiology, The First Affiliated Hospital of Guilin Medical University, Guilin, Guangxi China; 3 Medical Imaging Center, Affiliated Hospital of Shandong Second Medical University, Weifang, Shandong China; 4 Department of Radiology, Medical Imaging Institute of Tianjin, Tianjin First Central Hospital, School of Medicine, Nankai University, No. 24 Fu Kang Road, Nankai District, Tianjin, 300192 China; 5 Department of Radiology, The Affiliated Cancer Hospital of Xiangya School of Medicine, Central South University, Hunan Cancer Hospital, Changsha, China; 6 Department of Radiology, Tianjin Ninghe Hospital, Tianjin, China; 7 Department of Nuclear Medicine, Tianjin First Central Hospital, Tianjin, China; 8 First Central Hospital of Tianjin Medical University, Nankai District, Tianjin, China

**Keywords:** Neck adipose tissue, Inflammation, Aggressiveness, HPSCC, LSCC

## Abstract

**Background:**

The impact of neck adipose tissue (NAT) on the invasiveness of hypopharyngeal squamous cell carcinoma (HPSCC) and laryngeal squamous cell carcinoma (LSCC) remains uncertain. We investigated the roles of NAT and derived - neutrophil to lymphocyte ratio (dNLR) in the aggressiveness of HPSCC and LSCC, and established an adipose- inflammation-aggressiveness axis to identify high-risk factors.

**Methods:**

This retrospective study involved 412 patients with HPSCC or LSCC. Clinical characteristics, body mass index (BMI), NAT and dNLR were collected and calculated. Logistic regression models, restricted cubic splines (RCS) and mediation analysis were employed to evaluate the associations between NAT, dNLR and the aggressiveness of HPSCC and LSCC.

**Results:**

The cohort included 412 patients (mean age, 63 years; 93.69% male). Lower NAT was independently associated with advanced TNM stage (adjusted Odds Ratio [OR], 0.54; *p* = 0.015) and tumor local invasion (adjusted OR, 0.53; *p* = 0.008). Higher dNLR was significantly associated with advanced TNM stage (adjusted OR, 3.26; *p* < 0.001), lymph node metastasis (LNM) (adjusted OR, 1.40; *p* = 0.021), and tumor local invasion (adjusted OR, 2.29; *p* < 0.001). NAT showed a modest negative correlation with dNLR (*R* = -0.138, *p* = 0.005). Mediation analysis indicated that dNLR partially mediated the relationship between NAT and tumor aggressiveness.

**Conclusions:**

Reduced NAT is associated with increased tumor aggressiveness in HPSCC and LSCC, and this relationship may be partially mediated by elevated dNLR. The association appeared more pronounced in male patients. These findings suggest that local adiposity and inflammation may play a role in tumor behavior and warrant further investigation in future studies.

**Supplementary Information:**

The online version contains supplementary material available at 10.1186/s40644-025-00913-w.

## Introduction

Hypopharyngeal squamous cell carcinoma (HPSCC) and laryngeal squamous cell carcinoma (LSCC) are among the most aggressive malignancies of the head and neck region, often characterized by rapid local invasion, high recurrence rates, and poor survival outcomes [1, 2]. Despite advances in surgery, radiotherapy, and systemic therapies, clinical outcomes remain highly heterogeneous [3, 4], and many patients continue to face challenges such as treatment resistance and early recurrence. It is reported that underlying differences in tumor aggressiveness may play a critical role in prognosis [5]. However, current clinical tools lack the ability to capture such biological behavior accurately. Therefore, identifying novel and accessible biomarkers that reflect tumor aggressiveness is crucial for improving personalized risk stratification and treatment planning, thereby improving patient prognosis.

An increasing number of studies have recognized that tumor progression and invasiveness are influenced not only by intrinsic biological characteristics but also significantly by host-related factors, including body composition and systemic inflammation [6–8]. Adipose tissue is now recognized as a metabolically active organ that modulates immune responses and tumor behavior through the secretion of adipokines, cytokines, and microRNAs [9]. Importantly, adipose tissue is anatomically and functionally heterogeneous [10–12]. Different adipose regions—including abdominal visceral adipose tissue (VAT), subcutaneous adipose tissue (SAT), and other depots such as those in the neck, pericardium, liver, and muscle—may exhibit distinct biological properties. VAT is often considered pro-inflammatory and tumor-promoting [12], whereas SAT is generally regarded as neutral or even anti-inflammatory, potentially inhibiting tumor progression [13]. Chronic inflammation has been proposed as one of the biological mechanisms linking adiposity to high cancer risk [14]. Although several studies have explored the potential impacts of adipose tissue and inflammation markers, including derived - neutrophil to lymphocyte ratio (dNLR), on growth, development or prognosis of tumors [15–17], these studies primarily investigated the correlation between the two factors, yet few have explored whether systemic inflammation mediates the relationship between adipose tissue and tumor aggressiveness.

CT imaging is a valuable tool for quantifying diverse body composition and fat depots. CT-based adipose tissue has been extensively studied in relation to various cancers progression and prognosis, especially abdominal SAT and VAT [16–20]. It was reported that VAT was significantly correlated with disease progression-free survival and distant failure-free survival in patients with head and neck cancer [19]. However, as an important supporting structure surrounding head and neck tumors, research on neck adipose tissue (NAT) remains limited. To date, only one study has reported that higher NAT is associated with reduced mortality risk in patients with head and neck cancer [21]. But the impact of NAT on tumor behavior in head and neck cancer, especially in HPSCC and LSCC, is unknown.

This study hypothesizes that lower NAT is associated with tumor aggressiveness in HPSCC and LSCC, and that this association is partially mediated by systemic inflammation, as reflected by dNLR. The study aims to (1) assess the associations between NAT, dNLR, and tumor invasiveness; (2) examine the relationship between NAT and dNLR; and (3) evaluate the potential mediating effect of dNLR in the link between NAT and tumor aggressiveness. These findings may help establish a clinically relevant adipose–inflammation–aggressiveness axis, providing novel insight into personalized risk stratification for patients with HPSCC and LSCC.

## Material and method

### Ethics

This retrospective study was approved by the ethics committee of Tianjin First Central Hospital (2021N118KY). Meanwhile, the requirement for written informed consent was waived since the retrospective nature.

### Data collection

**Fig. 1:**
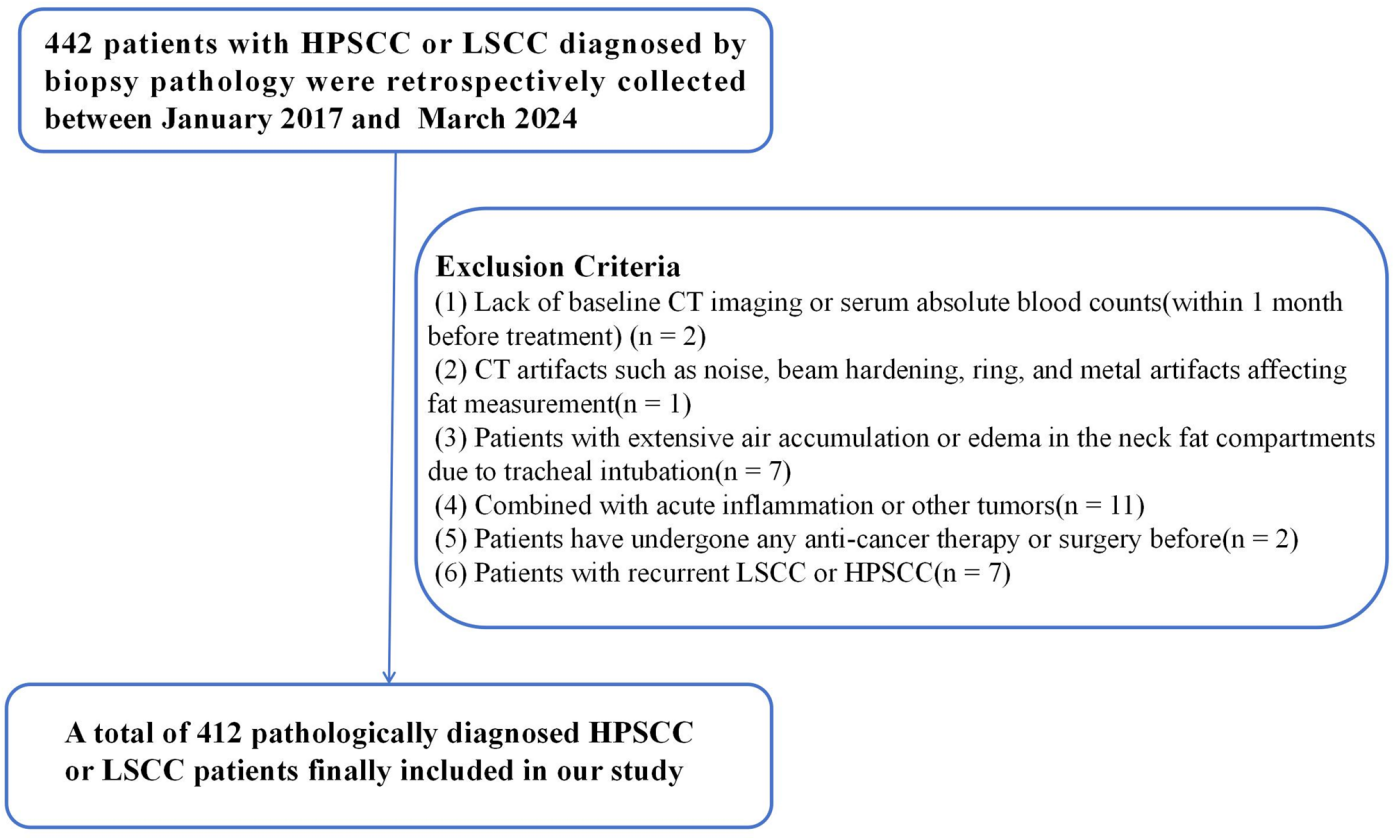
Flowchart of Patient Inclusion and Exclusion Criteria

A total of 412 patients with HPSCC or LSCC, diagnosed by biopsy at Tianjin First Central Hospital between January 2017 and March 2024, were retrospectively collected. The Exclusion criteria were shown in Fig. 1.

Baseline clinical data were collected included age, gender, weight, height, smoking history, alcohol history and serum absolute blood counts (leukocytes and neutrophils). Neck contrast-enhanced CT (CECT) scans were conducted prior to any systemic therapy.

dNLR was calculated as: neutrophils / (leukocytes - neutrophils) [22].

Body mass index (BMI) (kg/m²) was calculated as weight divided by height squared. BMI was categorized based on the WHO standards [23]: underweight (< 18.5 kg/m²), normal weight (18.5–24.9 kg/m²), overweight (25–29.9 kg/m²), and obesity (≥ 30 kg/m²).

### Imaging protocol

All patients underwent non-contrast and CECT scans before treatment within a week with a 64-slice spiral CT scanner (SOMATOM Definition; SIEMENS) or a 64-slice spiral CT scanner (SOMATOM Definition Flash; SIEMENS). The details of CT scanning parameters were provided in Appendix S1.

### The evaluation of aggressive characteristics of HPSCC and LSCC

The aggressive characteristics of HPSCC and LSCC included advanced stage, lymph node metastasis (LNM), and tumor local invasion. The clinical TNM stage was assessed according to the AJCC cancer staging manual [24]. TNM stages were categorized into early stage (stage I–II) or advanced stage (stage III–IV).

Patients were classified into two groups based on the presence or absence of LNM: non-LNM and LNM groups. The diagnostic criteria for LNM included: (1) surgery or biopsy; (2) evaluation based on the CECT images and at least one of the following criteria was met [25]: the short diameter larger than or equal to 10 mm, central necrosis or a contrast-enhancing rim, or evidence of extranodal extension [26].(Figure S1).

Patients were classified into two groups based on the presence or absence of invasion into neighboring structures by the primary tumor: non-tumor local invasion (non-invasion) group and tumor local invasion (invasion) group. Tumor local invasion included cartilage invasion and the extralaryngeal tumor spread. The diagnostic criteria for tumor local invasion were defined as follows: (1) Surgery or biopsy; (2) Evaluated based on CT images criteria [27]: (a) Thyroid cartilage invasion was considered present if erosion, lysis, or tumor spread through the cartilage was observed. Asymmetric sclerosis without erosion or lysis was negative, possibly indicated reactive changes. (b) For cricoid and arytenoid cartilages, invasion was considered if erosion, lysis, or asymmetric sclerosis in contact with the tumor was observed. (c) Extralaryngeal tumor spread was defined as extension of the primary tumor into surrounding soft tissues, including cervical and infrahyoid muscles, thyroid gland, esophagus, trachea, or deep lingual muscle, with or without cartilage penetration. (Figure S2)

TNM stage, LNM and tumor local invasion were evaluated by consensus between two experienced radiologists with 13 and 9 years of clinical experience in head and neck imaging, respectively. Any disagreements were discussed face-to-face and referred to a third senior expert if necessary until a consensus was reached. Kappa coefficient was used to estimate interobserver agreement. Kappa value >0.6 indicated good consistency.

### Measurement of adipose tissue

**Fig. 2:**
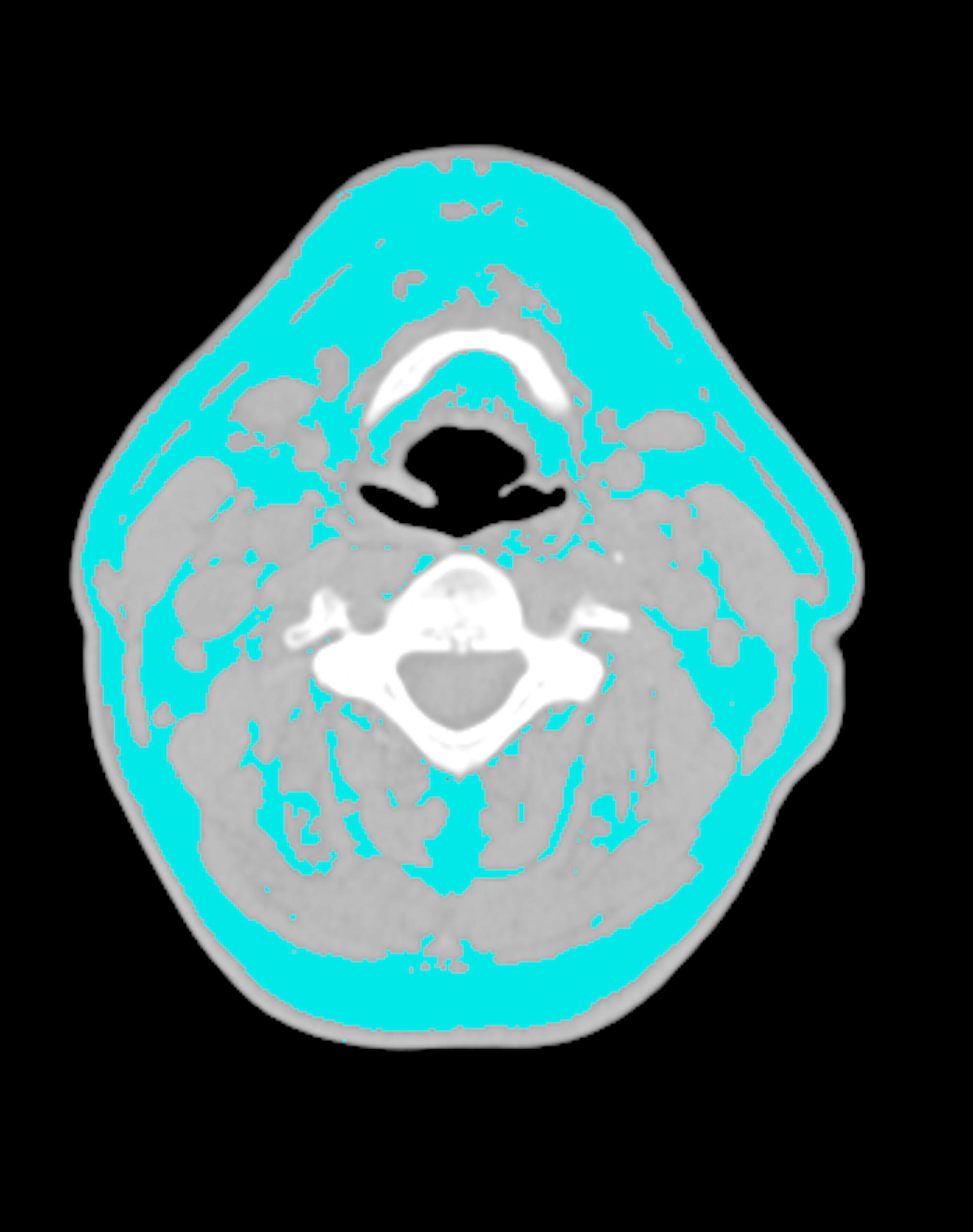
Measurement of NAT Area. Measurement of NAT using Slice-O-Matic V5.0 software (Tomovision, Montreal, Quebec, Canada) with standard thresholds of -190 HU to -30 HU. NAT was assessed by analyzing a single axial neck CT image at the level of the C4 vertebra transverse proces. NAT, neck adipose tissue

Area (cm^2^) of NAT was measured by analyzing a single axial neck CT image (Slice thickness: 1 mm) at the level of the C4 vertebra transverse process [28]. NAT was identified using Slice-O-Matic V5.0 software (Tomovision, Montreal, Quebec, Canada) with standard thresholds of -190 to -30 Hounsfield Unit (HU) (Fig. 2). A total of 150 images were randomly selected and measured twice by the same observer, with a one-month interval between the two measurements. The intra-observer correlation coefficient (ICC) was calculated. ICC > 0.8 indicated good agreement.

### Statistical analysis

Continuous variables were expressed as medians with interquartile ranges (IQR), and categorical variables were summarized as frequencies and percentages. Group comparisons were performed using the Mann–Whitney U test, χ² test, or Fisher’s exact test, as appropriate. Spearman’s rank correlation analysis was used to assess the association between variables. A two-sided *P* < 0.05 was considered statistically significant.

Univariable and multivariable binary logistic regression analyses were conducted to identify factors associated with tumor aggressiveness, with results reported as odds tatio (OR) and 95% confidence interval (CI).

RCS analysis was applied to assess potential nonlinear associations between NAT (as a continuous variable), dNLR and tumor aggressiveness. The spline function used piecewise cubic polynomials with linear constraints in the tails to ensure smooth fitting and reduce the influence of outliers [29, 30]. The optimal number of knots was determined based on the Akaike Information Criterion [30], and three knots were ultimately selected at the 10th, 50th, and 90th percentiles of the variable distribution.

Mediation analysis was performed to evaluate whether dNLR mediated the association between NAT and tumor aggressiveness. A linear regression model was first used to estimate the effect of NAT on dNLR (path a). Subsequently, a logistic regression model was fitted to estimate the effects of NAT and dNLR on tumor aggressive characteristics (path b and direct effect c′). Average causal mediation effects (ACME), average direct effects (ADE), total effects (c), and the proportion mediated were estimated. Nonparametric bootstrapping with 1,000 simulations was used for model inference [31].

All analyses were repeated in male patients to assess subgroup robustness. Statistical analyses were conducted using SPSS (version 26.0; IBM Corp., USA). Mediation and RCS were performed using R (version 4.3.0) with R “mediation” package and “rms” package. All figures were generated using OriginPro (version 2024; OriginLab Corp., USA).

## Results

### Patient characteristics

A total of 412 patients were included, comprising 158 (38.35%) with HPSCC and 254 (61.65%) with LSCC. Most patients were male (*n* = 386; 93.69%), and the median age at diagnosis was 63 years (IQR 57–68 years). A history of smoking was common, with 104 patients (25.24%) being former smokers and 252 patients (61.17%) current smokers. Alcohol consumption was noted in 199 current drinkers (48.30%) and 73 former drinkers (17.72%). Regarding BMI, most patients were categorized as normal weight (*n* = 244; 59.22%), followed by overweight (*n* = 119; 28.88%), underweight (*n* = 29; 7.04%), and obese (*n* = 20; 4.85%). Advanced TNM stage was observed in 299 (72.57%) patients, while LNM was present in 186 (45.15%). Local tumor invasion occurred in 174 patients (42.23%). Due to skewed distributions, NAT and dNLR were stratified using median values (NAT: 34 cm², dNLR: 1.56). NAT was grouped into High NAT and Low NAT based on the median. Details were provided in Table 1. Inter-observer reliability was excellent for NAT measurement (ICC = 0.993). Kappa values for inter-observer consistency in assessing TNM stage, LNM, and local tumor invasion ranged from 0.916 to 0.985 (Table S1).

**Table 1:** Baseline demographic and clinical characteristics

Variables	Total (*n* = 412)	Early stage (*n* = 113)	Advanced stage (*n* = 299)	Statistics	*P*
Age, M (Q_1_, Q_3_)	63.00 (57.00, 68.00)	63.00 (58.00, 68.00)	63.00 (56.00, 68.00)	Z=-0.68	0.496
Sex, n (%)				χ²=0.16	0.693
Male	386 (93.69)	105 (92.92)	281 (93.98)		
Female	26 (6.31)	8 (7.08)	18 (6.02)		
Smoking history, n (%)				χ²=9.66	0.008**
Never	56 (13.59)	25 (22.12)	31 (10.37)		
Ever	104 (25.24)	26 (23.01)	78 (26.09)		
Current	252 (61.17)	62 (54.87)	190 (63.55)		
Drinking history, n (%)				χ²=0.08	0.961
Never	140 (33.98)	38 (33.63)	102 (34.11)		
Ever	73 (17.72)	21 (18.58)	52 (17.39)		
Current	199 (48.30)	54 (47.79)	145 (48.49)		
BMI, n (%)				χ²=18.72	< 0.001***
Underweight	29 (7.04)	0 (0.00)	29 (9.70)		
Normal weight	244 (59.22)	61 (53.98)	183 (61.20)		
Overweight	119 (28.88)	44 (38.94)	75 (25.08)		
Obese	20 (4.85)	8 (7.08)	12 (4.01)		
NAT(Continuous), M (Q_1_, Q_3_)	34.00 (20.84, 45.05)	37.24 (29.19, 48.31)	32.54 (18.52, 43.95)	Z=-3.11	0.002**
NAT, n (%)				χ²=13.28	< 0.001***
Low NAT	206 (50.00)	40 (35.40)	166 (55.52)		
High NAT	206 (50.00)	73 (64.60)	133 (44.48)		
dNLR, M (Q_1_, Q_3_)	1.56 (1.22, 2.10)	1.31 (1.04, 1.58)	1.79 (1.34, 2.30)	Z=-6.48	< 0.001***
Tumor site, n (%)				χ²=25.73	< 0.001***
HPSCC	158 (38.35)	21 (18.58)	137 (45.82)		
LSCC	254 (61.65)	92 (81.42)	162 (54.18)		
TNM Stage					
T, n (%)				χ²=279.72	< 0.001***
T1	49 (11.89)	41 (36.28)	8 (2.68)		
T2	99 (24.03)	72 (63.72)	27 (9.03)		
T3	126 (30.58)	0 (0.00)	126 (42.14)		
T4	138 (33.50)	0 (0.00)	138 (46.15)		
N, n (%)				χ²=128.15	< 0.001***
N0	226 (54.85)	113 (100.00)	113 (37.79)		
N1	69 (16.75)	0 (0.00)	69 (23.08)		
N2	82 (19.90)	0 (0.00)	82 (27.42)		
N3	35 (8.50)	0 (0.00)	35 (11.71)		
M, n (%)				-	0.143
No	404 (98.06)	113 (100.00)	291 (97.32)		
Yes	8 (1.94)	0 (0.00)	8 (2.68)		

Z: Mann-Whitney test, χ²: Chi-square test, -: Fisher exact, M: Median, Q_1_: 1st Quartile, Q_3_: 3st Quartile, BMI body mass index, NAT neck adipose tissue, dNLR derived-Neutrophil to Lymphocyte Ratio

*P* < 0.05 (*), *P* < 0.01(**), *P* < 0.001(***)

### Association of BMI, NAT, and dNLR with TNM stage

**Fig. 3:**
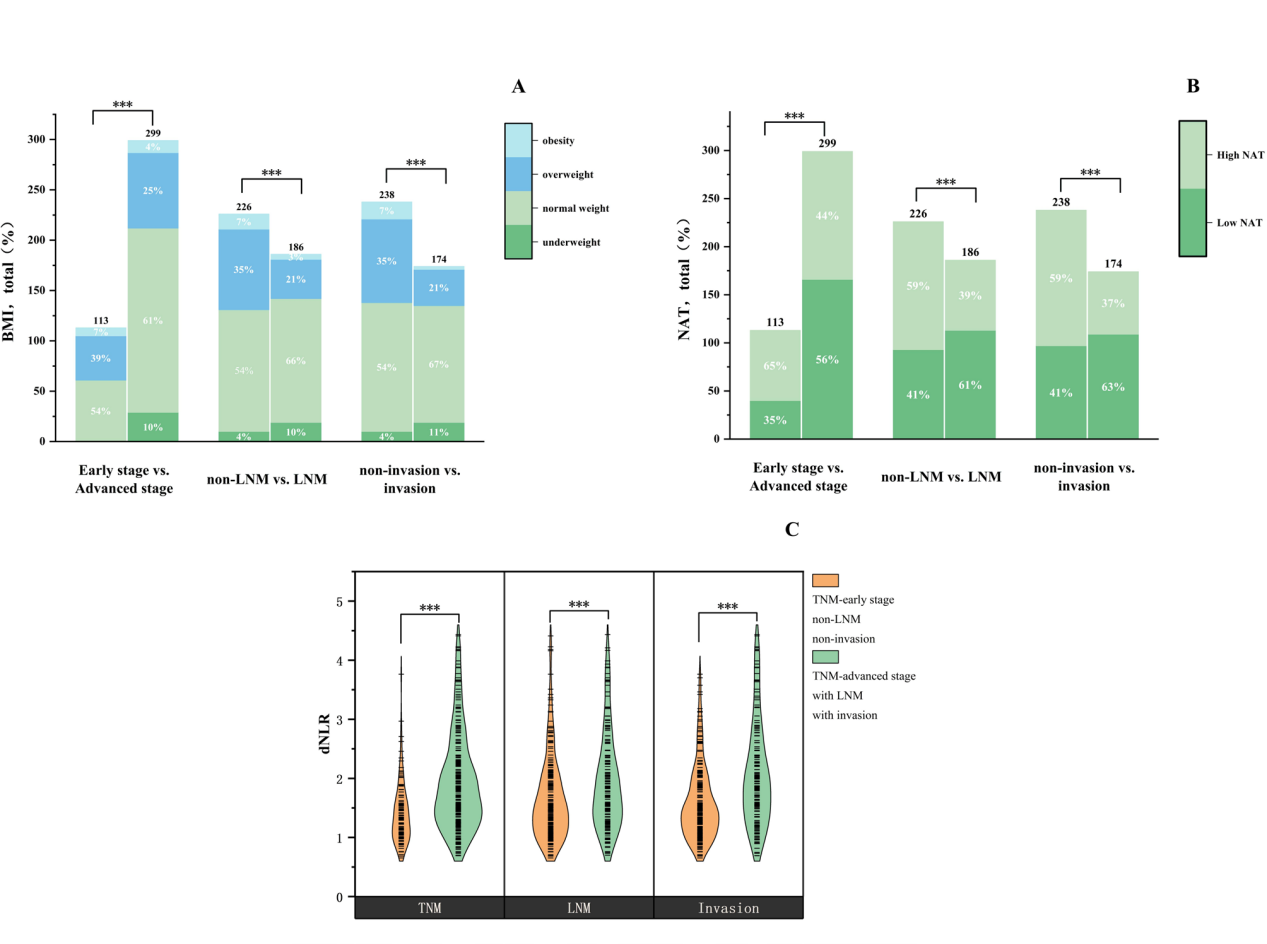
NAT(**A**), BMI(**B**) and dNLR(**C**) were statistically significant in both early and advanced stages, in non-LNM and LNM groups, and in non-invasion and invasion groups. BMI body mass index, NAT neck adipose tissue, dNLR derived-Neutrophil to Lymphocyte Ratio, LNM lymph node metastasis, Invasion, tumor local invasion. *P* < 0.05 (*), *P* < 0.01(**), *P* < 0.001(***)

Significant differences in NAT (*p* < 0.001), BMI (*p* < 0.001), and dNLR (*p* < 0.001) were noted between patients with early and advanced TNM stages. Patients in advanced stages exhibited higher dNLR [median 1.79 (IQR 1.34–2.30)] compared with early-stage patients [median 1.31 (IQR 1.04–1.58)]. High NAT occurred more frequently in early-stage patients (64.60%) than in advanced-stage patients (44.48%, *p* < 0.001). In the multivariable logistic regression adjusted for age, sex, tumor location, smoking and alcohol histories, higher dNLR was independently associated with advanced TNM stage (adjusted OR, 3.26; 95% CI, 2.09–5.10; *p* < 0.001), whereas higher NAT demonstrated an inverse association (adjusted OR, 0.54; 95% CI, 0.33–0.89; *p* = 0.015). BMI was not significantly associated with TNM stage in the adjusted analysis (Table S2, Fig. 3).

### Association of BMI, NAT, and dNLR with LNM

Patients with LNM had significantly higher dNLR values compared to those without LNM [median 1.76 (IQR 1.32–2.34) vs. 1.50 (IQR 1.12–2.01); *p* < 0.001]. Significant differences in NAT distribution were also observed between non-LNM and LNM groups (high NAT: 58.85% vs. 39.25%; *p* < 0.001). Additionally, BMI significantly differed between these groups (underweight: 4.42% vs. 10.22%; normal weight: 53.54% vs. 66.13%; overweight: 35.40% vs. 20.97%; obese: 6.64% vs. 2.69%; *p* < 0.001). Multivariable logistic regression revealed that higher dNLR remained independently associated with LNM (adjusted OR, 1.40; 95% CI, 1.05–1.86; *p* = 0.021). Despite significance in univariable analyses, neither NAT nor BMI retained independent associations with LNM after adjusting for confounders (Table S3, S4, Fig. 3).

### Association of BMI, NAT, and dNLR with tumor local invasion

Significant differences were identified in NAT, BMI, and dNLR between non-invasion and invasion groups (all *p* < 0.001). Multivariable analyses indicated that patients with local invasion had higher dNLR (adjusted OR, 2.29; 95% CI, 1.68–3.12; *p* < 0.001) and lower NAT (adjusted OR, 0.53; 95% CI, 0.33–0.85; *p* = 0.008). Furthermore, overweight (adjusted OR, 0.36; 95% CI, 0.13–0.96; *p* = 0.042) and obese patients (adjusted OR, 0.20; 95% CI, 0.04–0.97; *p* = 0.046) exhibited lower odds of local invasion compared to patients with normal BMI in adjusted models (Table S5, S6, Fig. 3).

### Non-linear associations between NAT, dNLR, and tumor aggressiveness

**Fig. 4:**
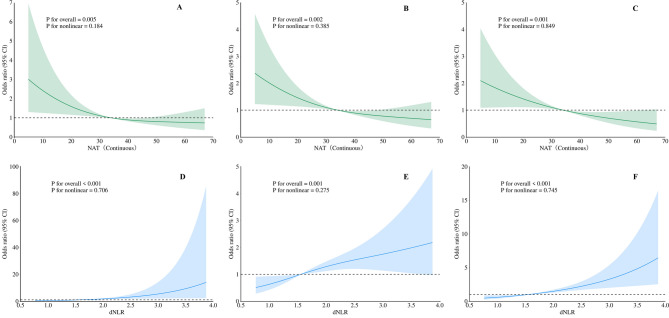
Nonlinear Association Between NAT, dNLR with Risk of Tumor Aggressiveness. RCS analysis for nonlinear trend of NAT (continuous) with the risk of TNM (**A**), LNM (**B**), tumor local invasion (**C**) and dNLR with the risk of TNM (**D**), LNM (**E**), tumor local invasion (**F**). All of *p* values for nonlinear were greater than 0.05, while *p* value for overall were less than 0.05, which indicated NAT (continuous) and dNLR demonstrated linear relationships with the risk of aggressive characteristics of HPSCC and LSCC. NAT neck adipose tissue, dNLR derived-Neutrophil to Lymphocyte Ratio, LNM lymph node metastasis. CI, confidence interval. *P* < 0.05 (*), *P* < 0.01(**), *P* < 0.001(***)

Restricted cubic spline (RCS) analyses revealed linear associations of dNLR and NAT with tumor aggressiveness, showing no evidence of significant non-linear relationships (*p* for nonlinear > 0.05). dNLR positively correlated with advanced TNM stage (*p* for overall = 0.005), LNM (*p* for overall = 0.002), and tumor local invasion (*p* for overall = 0.001). Conversely, NAT exhibited negative linear relationships with advanced stage (*p* for overall < 0.001), LNM (*p* for overall = 0.001), and local invasion (*p* for overall < 0.001) (Fig. 4).

### Relationship between NAT and dNLR

Significantly higher dNLR values were found in patients with low NAT compared to high NAT [median 1.66 (IQR 1.27–2.15) vs. 1.50 (IQR 1.16–2.06); *p* = 0.018]. NAT as a continuous variable negatively correlated with dNLR (*R* = -0.138; *p* = 0.005).

### Mediation analysis

**Fig. 5:**
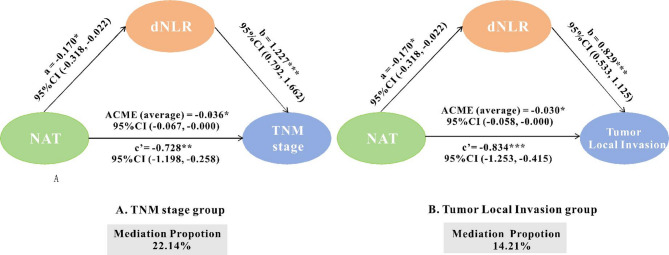
Effect of NAT on Tumor Aggressiveness is Partially Mediated by dNLR. Mediation analysis was conducted to investigate the effect of NAT on TNM stage (**A**), Tumor local invasion (**B**) with mediation through dNLR. The effects of NAT on tumor invasiveness were partially mediated by dNLR. A estimated from linear regression; b and c’ from logistic regression (log odds); ab from mediation analysis. dNLR derived-Neutrophil to Lymphocyte Ratio, NAT neck adipose tissue. *P* < 0.05 (*), *P* < 0.01(**), *P* < 0.001(***)

Mediation analyses were conducted for tumor aggressive characteristics including TNM stage and tumor local invasion. For TNM, the ACME (average) was − 0.036 (95% CI: -0.067, -0.00, *p* = 0.028), and ADE (average) was − 0.126 (95% CI: -0.200, -0.040, *p* = 0.002). The total effect was − 0.161 (95% CI: -0.240, -0.069, *p* < 0.001), with 22.14% of the total effect mediated via dNLR. For tumor local invasion, the ACME (average) was − 0.030 (95% CI: -0.058, 0.000, *p* = 0.036), ADE (average) was − 0.183 (95% CI: -0.277, -0.100, *p* < 0.001), the total effect was − 0.214 (95% CI: -0.310, -0.120, *p* < 0.001), and 14.21% of the effect was mediated. These results indicated that dNLR partially mediated the effect of NAT on both tumor invasion and TNM stage. (Fig. 5)

### Subgroup analysis stratified by gender

As HPSCC and LSCC are more prevalent in men, we investigated the potential impacts of BMI, NAT and dNLR on aggressive characteristics of HPSCC and LSCC in male. NAT was re-stratified based on the median (33.73 cm^2^), grouping into low and high NAT groups. In the male subgroup (*n* = 386), high dNLR remained significantly associated with advanced stage (adjusted OR, 3.15; 95% CI, 2.00–4.97, *p* < 0.001), LNM (adjusted OR, 1.46; 95% CI, 1.09–1.98, *p* = 0.013), and tumor local invasion (adjusted OR, 2.16; 95% CI, 1.58–2.95, *p* < 0.001). NAT was not only associated with advanced stage (adjusted OR, 0.56; 95% CI, 0.33–0.93, *p* = 0.024) and tumor local invasion (adjusted OR, 0.60; 95% CI, 0.37–0.96, *p* = 0.034), but also exhibited a relationship with LNM (adjusted OR, 0.60; 95% CI, 0.37–0.99, *p* = 0.046), which demonstrated a stronger relationship with the aggressive characteristics to the all-gender cohort. In contrast, we did not find any association between BMI and the aggressive characteristics of HPSCC and LSCC in multivariable-adjusted analyses (Tables S7–S12, Figure S3).

## Discussion

To our knowledge, this study is the first to explore the interrelationship between NAT, dNLR, and tumor aggressiveness in patients with HPSCC and LSCC using a mediation model. We identified the following key findings: First, decreased NAT was independently associated with advanced TNM stage and tumor local invasion, while elevated dNLR was positively associated with tumor aggressive characteristics. This finding suggests that reduced cervical adiposity and heightened systemic inflammation may serve as independent markers of tumor progression. Second, NAT was inversely correlated with dNLR, indicating that patients with lower NAT tended to have higher levels of systemic inflammation. Third, mediation analysis revealed that dNLR partially mediated the relationship between NAT and tumor aggressiveness, suggesting that systemic inflammation may play a role in linking local adiposity and tumor progression. These results support a preliminary conceptual framework of an adipose–inflammation–aggressiveness axis.

In our study, there was an inverse association between NAT and aggressive characteristics of HPSCC and LSCC, including TNM stage and tumor local invasion. These results were consistent with previous studies [18, 21], which reported that the VAT was significantly lower in those with metastatic status comparing to those with localized renal cell carcinoma [18], and higher cervical adiposity was connected to reduced mortality risk in patients with head and neck cancer [21]. While obesity was generally considered to be associated with an increased risk of various cancers [32], some evidence suggests that specific types of adipose tissue may also play a role in suppressing tumor progression and adverse outcomes through mechanisms such as immune regulation and clonal selection—a phenomenon known as the “obesity paradox” [33, 34].

One possible explanation is that NAT reflects adipose tissue with distinct metabolic and immune phenotypes. The cervical region contains not only superficial subcutaneous fat but also metabolically active brown adipose tissue, which may influence systemic inflammation and the tumor microenvironment (TME) via secretion of adiponectin, IL-10, and FGF21 [35–37]. Adiponectin has been shown to inhibit tumor cell proliferation through activation of the AMPK–PPARα signaling pathway, IL-10 suppresses proinflammatory cytokine expression, and FGF21 plays a role in modulating oxidative stress and immune homeostasis. Collectively, these factors may create an anti-inflammatory, metabolically favorable microenvironment that limits tumor progression. Additionally, superficial adipocytes can secrete thrombospondin-1 (TSP-1), which contributes to extracellular matrix remodeling and inhibition of angiogenesis, further shaping a tumor-suppressive TME [38]. On the other hand, reduced NAT may not only precede but also result from aggressive tumor biology. For instance, cancer cachexia—a common metabolic disorder in advanced malignancies—often leads to rapid depletion of both fat and skeletal muscle mass, resulting in a marked reduction of adipose tissue [39]. However, while high NAT was associated with reduced risk of LNM in univariable analyses, this association was no longer significant in multivariable analysis after adjusting for covariates. It is possible that the interplay of multiple factors attenuated this association.

Regarding inflammation, we found that elevated dNLR was significantly associated with tumor aggressiveness. In various malignancies, including head and neck cancer, systemic inflammatory markers have been shown to be closely associated with increased tumor aggressiveness and poorer prognosis [40–43]. Elevated dNLR, particularly driven by an increase in neutrophil counts, has been implicated in multiple protumorigenic processes [44, 45]. Recent evidence demonstrates that tumor-infiltrating neutrophils in LSCC exhibit high PD-L1 expression and potently suppress CD8⁺ T cell activity, thereby facilitating tumor immune evasion and progression [45].

Furthermore, we observed a significant negative association between NAT and dNLR, suggesting that decreased NAT may be linked to heightened systemic inflammation and may possess anti-inflammatory properties similar to SAT [13]. This finding is consistent with previous studies that reported lower systemic inflammation and better cancer outcomes in association with higher SAT levels [15]. The enhanced inflammatory state observed in patients with lower NAT may be attributed to a reduction in anti-inflammatory adipokine secretion or metabolic dysregulation induced by tumor burden [35–37].

Based on the above findings, we further conducted a mediation analysis. Mediation analysis suggested that dNLR may act as an intermediary in the relationship between NAT and tumor aggressiveness, forming a potential “NAT–inflammation–tumor aggressiveness” axis. To our knowledge, this is the first study to statistically validate this mediation pathway in patients with HPSCC and LSCC. Although mediation effects do not imply causality, and the retrospective design of this study further limits causal inference [31], the identified statistical pathway integrates local adipose distribution, systemic inflammation, and tumor progression, with a biologically plausible interpretability. The mediation results provide preliminary statistical support for this hypothesized NAT–inflammation–tumor aggressiveness axis, indicating that cervical adipose tissue may influence tumor biology in HPSCC and LSCC indirectly through modulation of systemic inflammation. This finding lays a theoretical foundation and research direction for future studies focusing on regional fat evaluation and anti-inflammatory therapeutic strategies, and warrants further validation through prospective cohorts and mechanistic investigations. Notably, the mediation effect of dNLR was more pronounced for TNM stage than for local tumor invasion (22.14% vs. 14.21%), possibly because TNM stage reflects a broader spectrum of tumor progression, encompassing local invasion, regional lymph node metastasis, and distant spread as systemic features. Since dNLR reflects systemic inflammatory and immunosuppressive states, its mediating role may be more relevant to the overall advancement of tumor burden than to localized invasion alone.

Gender-specific subgroup analysis revealed a significant inverse relationship between NAT and LNM in males, whereas such an association was absent in the overall cohort. This discrepancy may reflect gender-related differences in adipose tissue distribution and metabolic function, suggesting further research may be needed to clarify gender-specific adiposity effects.

The association between BMI and tumor aggressiveness appeared weaker in our study. Only overweight or obese patients demonstrated a decreased risk of tumor local invasion in adjusted analyses, which partly concurred with prior studies indicating BMI-related protective effects against tumor invasion [46, 47]. However, the relatively weaker associations observed in our study reflect inherent limitations of BMI as a general indicator of adiposity, as it failed to differentiate fat from muscle mass or accurately reflect local fat distribution. Additionally, adjustment for multiple confounders might have diminished the apparent influence of BMI, which indicated the need for more precise, localized measures of adiposity.

Our study has several limitations. First, TNM staging was based on clinical rather than pathological data, which may have introduced misclassification and affected the accuracy of aggressiveness assessment. Second, as a single-center study, external generalizability is limited due to potential institutional and population-specific factors. Third, the retrospective design precluded analysis of pre-diagnostic changes in adiposity and inflammation, limiting our ability to address reverse causality. Fourth, the mediation results support a hypothetical pathway, but do not imply causality. Future studies using causal inference approaches, such as Mendelian randomization, are needed to validate these relationships. Lastly, total NAT was measured without distinguishing superficial from deep fat compartments, which may have different biological roles. Future studies should incorporate detailed fat compartment analysis.

In conclusion, decreased NAT was associated with increased tumor aggressiveness in HPSCC and LSCC, and this association was partially mediated by elevated dNLR. These findings suggest that cervical adiposity may influence tumor behavior both directly and indirectly through inflammatory pathways. The observed sex-specific differences further support the complexity of host-tumor interactions. Given the limitations of BMI, NAT quantification on routine imaging may offer a more precise, non-invasive tool for risk stratification. Future studies are required to validate these associations and explore the potential of adipose–inflammation–aggressiveness axis as therapeutic targets.

## Electronic supplementary material

Below is the link to the electronic supplementary material.

Supplementary Material 1

Supplementary Material 2

Supplementary Material 3

Supplementary Material 4

Supplementary Material 5

Supplementary Material 6

Supplementary Material 7

Supplementary Material 8

Supplementary Material 9

Supplementary Material 10

Supplementary Material 11

Supplementary Material 12

Supplementary Material 13

Supplementary Material 14

Supplementary Material 15

Supplementary Material 16

Supplementary Material 17

## Data Availability

The data that support the findings of this study are available from the corresponding author upon reasonable request.

## Abbreviations

ACME: Average causal mediation effects
ADE: Average direct effects
BMI: Body mass index
CI: Confidence intervals
CECT: Contrast-enhanced CT
dNLR: Derived - neutrophil to lymphocyte ratio
HPSCC: Hypopharyngeal squamous cell carcinoma
ICC: The intra-observer correlation coefficient
IQR: Interquartile ranges
LNM: Lymph node metastasis
LSCC: Laryngeal squamous cell carcinoma
NAT: Neck adipose tissue
OR: Odds ratio
RCS: Restricted cubic splines
SAT: Subcutaneous adipose tissue
VAT: Visceral adipose tissue

## References

[1] Sung H, Ferlay J, Siegel RL, Laversanne M, Soerjomataram I, Jemal A, Bray F. Global cancer statistics 2020: GLOBOCAN estimates of incidence and mortality worldwide for 36 cancers in 185 countries. CA Cancer J Clin. 2021;71:209–49. 33538338 10.3322/caac.21660

[2] Steuer CE, El-Deiry M, Parks JR, Higgins KA, Saba NF. An update on larynx cancer. CA Cancer J Clin. 2017;67:31–50. 27898173 10.3322/caac.21386

[3] Lin C-H, Yan J-L, Yap W-K, et al. Prognostic value of interim CT-based peritumoral and intratumoral radiomics in laryngeal and hypopharyngeal cancer patients undergoing definitive radiotherapy. Radiother Oncol J Eur Soc Ther Radiol Oncol. 2023;189:109938. 10.1016/j.radonc.2023.109938 37806562

[4] Tsai T-Y, Yap W-K, Wang T-H, Lu Y-A, See A, Hu Y-F, Huang Y, Kao H-K, Chang K-P. Upfront surgery versus upfront concurrent chemoradiotherapy as primary modality in hypopharyngeal squamous cell carcinoma: a systematic review and meta-analysis. J Otolaryngol - Head Neck Surg J Oto-Rhino-Laryngol Chir Cervicofac. 2024;53:19160216241293633. 10.1177/19160216241293633 PMC11528607 39468833

[5] Leemans CR, Snijders PJF, Brakenhoff RH. The molecular landscape of head and neck cancer. Nat Rev Cancer. 2018;18:269–82. 29497144 10.1038/nrc.2018.11

[6] Anderson LJ, Lee J, Anderson B, et al. Whole-body and adipose tissue metabolic phenotype in cancer patients. J Cachexia Sarcopenia Muscle. 2022;13:1124–33. 35088949 10.1002/jcsm.12918 PMC8977952

[7] Li K, Zeng X, Liu P, Zeng X, Lv J, Qiu S, Zhang P. The role of Inflammation-Associated factors in head and neck squamous cell carcinoma. J Inflamm Res. 2023;16:4301–15. 37791117 10.2147/JIR.S428358 PMC10544098

[8] Moukarzel LA, Ferrando L, Stylianou A, et al. Impact of obesity and white adipose tissue inflammation on the omental microenvironment in endometrial cancer. Cancer. 2022;128:3297–309. 35793549 10.1002/cncr.34356 PMC9976596

[9] Avgerinos KI, Spyrou N, Mantzoros CS, Dalamaga M. Obesity and cancer risk: emerging biological mechanisms and perspectives. Metabolism. 2019;92:121–35. 30445141 10.1016/j.metabol.2018.11.001

[10] Song YC, Lee SE, Jin Y, Park HW, Chun K-H, Lee H-W. Classifying the linkage between adipose tissue inflammation and tumor growth through Cancer-Associated adipocytes. Mol Cells. 2020;43:763–73. 32759466 10.14348/molcells.2020.0118 PMC7528682

[11] Fox CS, Massaro JM, Hoffmann U, et al. Abdominal visceral and subcutaneous adipose tissue compartments: association with metabolic risk factors in the Framingham heart study. Circulation. 2007;116:39–48. 17576866 10.1161/CIRCULATIONAHA.106.675355

[12] Doyle SL, Donohoe CL, Lysaght J, Reynolds JV. Visceral obesity, metabolic syndrome, insulin resistance and cancer. Proc Nutr Soc. 2012;71:181–9. 22051112 10.1017/S002966511100320X

[13] Hwang I, Jo K, Shin KC, et al. GABA-stimulated adipose-derived stem cells suppress subcutaneous adipose inflammation in obesity. Proc Natl Acad Sci U S A. 2019;116:11936–45. 31160440 10.1073/pnas.1822067116 PMC6575165

[14] Wang Y, Zhu N, Zhang C, Wang Y, Wu H, Li Q, Du K, Liao D, Qin L. Friend or foe: multiple roles of adipose tissue in cancer formation and progression. J Cell Physiol. 2019;234:21436–49. 31054175 10.1002/jcp.28776

[15] He M, Chen Z-F, Zhang L, et al. Associations of subcutaneous fat area and systemic Immune-inflammation index with survival in patients with advanced gastric cancer receiving dual PD-1 and HER2 Blockade. J Immunother Cancer. 2023;11:e007054. 37349127 10.1136/jitc-2023-007054 PMC10314655

[16] Willemsen ACH, De Moor N, Van Dessel J, et al. The predictive and prognostic value of weight loss and body composition prior to and during immune checkpoint Inhibition in recurrent or metastatic head and neck cancer patients. Cancer Med. 2022;12:7699–712. 36484469 10.1002/cam4.5522 PMC10134381

[17] He W-Z, Jiang C, Liu L-L, et al. Association of body composition with survival and inflammatory responses in patients with non-metastatic nasopharyngeal cancer. Oral Oncol. 2020;108:104771. 32485608 10.1016/j.oraloncology.2020.104771

[18] Tan C-C, Sheng T-W, Chang Y-H, Wang L-J, Chuang C-K, Wu C-T, Pang S-T, Shao I-H. Utilizing computed tomography to analyze the morphomic change between patients with localized and metastatic renal cell carcinoma: body composition varies according to Cancer stage. J Clin Med. 2022;11:4444. 35956059 10.3390/jcm11154444 PMC9369886

[19] Lee JW, Ban MJ, Park JH, Lee SM. Visceral adipose tissue volume and CT-attenuation as prognostic factors in patients with head and neck cancer. Head Neck. 2019;41:1605–14. 30636185 10.1002/hed.25605

[20] Demirel E, Dilek O. A new finding for the obesity paradox? Evaluation of the relationship between muscle and adipose tissue in nuclear grade prediction in patients with clear cell renal cell carcinoma. Acta Radiol Stockh Swed 1987. 2023;64:1659–67. 10.1177/02841851221126358 37023029

[21] Carrilho LAO, Juliani FL, de Moreira RC L, et al. Adipose tissue characteristics as a new prognosis marker of patients with locally advanced head and neck cancer. Front Nutr. 2025;12:1472634. 40161297 10.3389/fnut.2025.1472634 PMC11949816

[22] Ou Y, Liang S, Gao Q, Shang Y, Liang J, Zhang W, Liu S. Prognostic value of inflammatory markers NLR, PLR, LMR, dNLR, ANC in melanoma patients treated with immune checkpoint inhibitors: a meta-analysis and systematic review. Front Immunol. 2024;15:1482746. 39493767 10.3389/fimmu.2024.1482746 PMC11527641

[23] World Health Organization. Obesity: preventing and managing the global epidemic. Report of a WHO consultation. World Health Organ Tech Rep Ser. 2000;894:i–xii, 1–253. 11234459

[24] Amin MB, Edge SB, Greene FL, Byrd DR, Brookland RB, Washington MK, Gershenwald JE. AJCC Cancer staging manual. 8th ed. New York: Springer; 2017.

[25] Hoang JK, Vanka J, Ludwig BJ, Glastonbury CM. Evaluation of cervical lymph nodes in head and neck cancer with CT and MRI: tips, traps, and a systematic approach. AJR Am J Roentgenol. 2013;200:W17–25. 23255768 10.2214/AJR.12.8960

[26] Henson C, Abou-Foul AK, Yu E, et al. Criteria for the diagnosis of extranodal extension detected on radiological imaging in head and neck cancer: head and neck Cancer international group consensus recommendations. Lancet Oncol. 2024;25:e297–307. 38936388 10.1016/S1470-2045(24)00066-4

[27] Kuno H, Onaya H, Iwata R, Kobayashi T, Fujii S, Hayashi R, Otani K, Ojiri H, Yamanaka T, Satake M. Evaluation of cartilage invasion by laryngeal and hypopharyngeal squamous cell carcinoma with dual-energy CT. Radiology. 2012;265:488–96. 22984188 10.1148/radiol.12111719

[28] Li H-X, Zhang F, Zhao D, et al. Neck circumference as a measure of neck fat and abdominal visceral fat in Chinese adults. BMC Public Health. 2014;14:311. 24708638 10.1186/1471-2458-14-311 PMC4004507

[29] Yap W-K, Shih M-C, Kuo C, Pai P-C, Chou W-C, Chang K-P, Tsai M-H, Tsang N-M. Development and validation of a nomogram for assessing survival in patients with metastatic lung cancer referred for radiotherapy for bone metastases. JAMA Netw Open. 2018;1:e183242. 30646236 10.1001/jamanetworkopen.2018.3242 PMC6324455

[30] Harrell FE Jr. Regression modeling strategies. 2nd ed. New York: Springer; 2015.

[31] Tingley D, Yamamoto T, Hirose K, Keele L, Imai K. Mediation: R package for causal mediation analysis. J Stat Softw. 2014;59:1–38.26917999

[32] Sung H, Siegel RL, Torre LA, et al. Global patterns in excess body weight and the associated cancer burden. CA Cancer J Clin. 2019;69:88–112. 30548482 10.3322/caac.21499

[33] Quail DF, Dannenberg AJ. The obese adipose tissue microenvironment in cancer development and progression. Nat Rev Endocrinol. 2019;15:139–54. 30459447 10.1038/s41574-018-0126-x PMC6374176

[34] Simati S, Kokkinos A, Dalamaga M, Argyrakopoulou G. Obesity paradox: fact or fiction? Curr Obes Rep. 2023;12:75–85. 36808566 10.1007/s13679-023-00497-1

[35] Cypess AM. Reassessing human adipose tissue. N Engl J Med. 2022;386:768–79. 35196429 10.1056/NEJMra2032804

[36] Ghesmati Z, Rashid M, Fayezi S, Gieseler F, Alizadeh E, Darabi M. An update on the secretory functions of brown, white, and beige adipose tissue: towards therapeutic applications. Rev Endocr Metab Disord. 2024;25:279–308. 38051471 10.1007/s11154-023-09850-0 PMC10942928

[37] Kounatidis D, Vallianou NG, Karampela I, Grivakou E, Dalamaga M. The intricate role of adipokines in cancer-related signaling and the tumor microenvironment: insights for future research. Semin Cancer Biol. 2025;113:130–50. 40412490 10.1016/j.semcancer.2025.05.013

[38] Lengyel E, Makowski L, DiGiovanni J, Kolonin MG. Cancer as a matter of fat: the crosstalk between adipose tissue and tumors. Trends Cancer. 2018;4:374–84. 29709261 10.1016/j.trecan.2018.03.004 PMC5932630

[39] Arends J, Bachmann P, Baracos V, et al. ESPEN guidelines on nutrition in cancer patients. Clin Nutr Edinb Scotl. 2017;36:11–48. 10.1016/j.clnu.2016.07.015 27637832

[40] Mezquita L, Preeshagul I, Auclin E et al. (2021) Predicting immunotherapy outcomes under therapy in patients with advanced NSCLC using dNLR and its early dynamics. Eur J Cancer Oxf Engl 1990 151:211–220. 10.1016/j.ejca.2021.03.011 34022698

[41] Winarto H, Habiburrahman M, Anggraeni TD, Nuryanto KH, Julianti RA, Purwoto G, Andrijono A. The utility of Pre-Treatment inflammation markers as associative factors to the adverse outcomes of vulvar cancer: A study on staging, nodal involvement, and metastasis models. J Clin Med. 2023;12:96. 10.3390/jcm12010096 PMC9821387 36614896

[42] Ma SJ, Yu H, Khan M, et al. Evaluation of optimal threshold of neutrophil-lymphocyte ratio and its association with survival outcomes among patients with head and neck cancer. JAMA Netw Open. 2022;5:e227567. 35426920 10.1001/jamanetworkopen.2022.7567 PMC9012962

[43] Qi H. Role and research progress of hematological markers in laryngeal squamous cell carcinoma. Diagn Pathol. 2023;18:50. 37081512 10.1186/s13000-023-01335-7 PMC10120220

[44] Jaillon S, Ponzetta A, Di Mitri D, Santoni A, Bonecchi R, Mantovani A. Neutrophil diversity and plasticity in tumour progression and therapy. Nat Rev Cancer. 2020;20:485–503. 32694624 10.1038/s41568-020-0281-y

[45] Tang D, Zhang D, Heng Y, Zhu X-K, Lin H-Q, Zhou J, Tao L, Lu L-M. Tumor-infiltrating PD-L1 + neutrophils induced by GM-CSF suppress T cell function in laryngeal squamous cell carcinoma and predict unfavorable prognosis. J Inflamm Res. 2022;15:1079–97. 35210813 10.2147/JIR.S347777 PMC8859980

[46] Choi JS, Kim E-K, Moon HJ, Kwak JY. Higher body mass index May be a predictor of extrathyroidal extension in patients with papillary thyroid microcarcinoma. Endocrine. 2015;48:264–71. 24858734 10.1007/s12020-014-0293-z

[47] Li CL, Dionigi G, Zhao YS, Liang N, Sun H. Influence of body mass index on the clinicopathological features of 13,995 papillary thyroid tumors. J Endocrinol Invest. 2020;43:1283–99. 32166701 10.1007/s40618-020-01216-6

