# Supplementary material for: Inflammation as a mediator between neck adipose tissue and tumor aggressiveness in hypopharyngeal and laryngeal squamous cell carcinoma
Source: Cancer Imaging. 2025 Jul 29;25:95. doi: 10.1186/s40644-025-00913-w (PMC12309162; doi:10.1186/s40644-025-00913-w)
Supplement: Supplementary file 1 — Supplementary Material 1 [file 40644_2025_913_MOESM1_ESM.docx]

**Supplementary appendix S1**

All patients underwent CECT before treatment within a week with a 64-slice spiral CT scanner (SOMATOM Definition; SIEMENS) or a 64-slice spiral CT scanner (SOMATOM Definition Flash; SIEMENS). The CT scanning parameters were as follows: 120 kV; 165–200 mAs; a field of view (FOV) of 200 mm; a matrix of 512 × 512; and helical thickness of 5 mm. Contrast-enhanced scanning were performed after non-contrast CT scanning. For contrast-enhanced scanning, 60–70 ml of an iodinated nonionic contrast agent (iohexol; 350 mg/dl iodine, SOMATOM Definition Flash, Siemens Healthineers AG) was administered through the right elbow median vein by a pump injector. The dosage was 1 ml/kg with a flow rate of 3 ml/s. The enhanced scanning delay time was 60 s.
