# Supplementary material for: Inflammation as a mediator between neck adipose tissue and tumor aggressiveness in hypopharyngeal and laryngeal squamous cell carcinoma
Source: Cancer Imaging. 2025 Jul 29;25:95. doi: 10.1186/s40644-025-00913-w (PMC12309162; doi:10.1186/s40644-025-00913-w)
Supplement: Supplementary file 2 — Supplementary Material 2 [file 40644_2025_913_MOESM2_ESM.docx]

**Supplementary Table 1 The consistency of inter-observer**

|  | K / ICC | 95% CI | SE | *p* |
| --- | --- | --- | --- | --- |
| TNM stage | ^a^ 0.963 | 0.941 - 0.985 | 0.011 | 0.000*** |
| LNM | ^a^ 0.916 | 0.881- 0.950 | 0.018 | 0.000*** |
| Tumor local invasion | ^a^ 0.985 | 0.968 - 1.002 | 0.009 | 0.000*** |
| NAT | ^b^ 0.993 | 0.990 ~ 0.995 |  |  |
| * p<0.05 ** p<0.01 *** p<0.001; ^a^ Cohen's kappa; ^b^ICC intra-observer correlation coefficient; LNM Lymph node metastasis; CI, confidence interval; NAT neck adipose tissue | | | | |
