# Supplementary material for: Inflammation as a mediator between neck adipose tissue and tumor aggressiveness in hypopharyngeal and laryngeal squamous cell carcinoma
Source: Cancer Imaging. 2025 Jul 29;25:95. doi: 10.1186/s40644-025-00913-w (PMC12309162; doi:10.1186/s40644-025-00913-w)
Supplement: Supplementary file 3 — Supplementary Material 3 [file 40644_2025_913_MOESM3_ESM.docx]

**Supplementary Table 2. Univariable and multivariable analyses for TNM stage (n=412)**

| Variables | Univariable analysis | | | | |  | Multivariable analysis | | | | |
| --- | --- | --- | --- | --- | --- | --- | --- | --- | --- | --- | --- |
|  | β | S.E | Z | *P* | OR (95%CI) |  | β | S.E | Z | *P* | Adjusted OR (95%CI) |
| BMI |  |  |  |  |  |  |  |  |  |  |  |
| Underweight |  |  |  |  | 1.00 (Reference) |  |  |  |  |  |  |
| Normal weight | -16.47 | 734.64 | -0.02 | 0.982 | 0.00 (0.00 ~ Inf) |  |  |  |  |  |  |
| Overweight | -17.03 | 734.64 | -0.02 | 0.982 | 0.00 (0.00 ~ Inf) |  |  |  |  |  |  |
| Obese | -17.16 | 734.64 | -0.02 | 0.981 | 0.00 (0.00 ~ Inf) |  |  |  |  |  |  |
| NAT |  |  |  |  |  |  |  |  |  |  |  |
| Low NAT |  |  |  |  | 1.00 (Reference) |  |  |  |  |  | 1.00 (Reference) |
| High NAT | -0.82 | 0.23 | -3.60 | <0.001*** | 0.44 (0.28 ~ 0.69) |  | -0.61 | 0.25 | -2.43 | 0.015** | 0.54 (0.33 ~ 0.89) |
| dNLR | 1.27 | 0.22 | 5.73 | <0.001*** | 3.56 (2.31 ~ 5.50) |  | 1.18 | 0.23 | 5.18 | <0.001*** | 3.26 (2.09 ~ 5.10) |
| Dependent variable: TNM stage, Adjusted covariates: sex, age, tumor site, smoking history, drinking history, BMI body mass index, NAT neck adipose tissue, dNLR derived-Neutrophil to Lymphocyte Ratio  OR: Odds Ratio, CI: Confidence Interval, *P*<0.05 (*), *P*< 0.01(**), *P*< 0.001(***) | | | | | | | | | | | |
