# Supplementary material for: Inflammation as a mediator between neck adipose tissue and tumor aggressiveness in hypopharyngeal and laryngeal squamous cell carcinoma
Source: Cancer Imaging. 2025 Jul 29;25:95. doi: 10.1186/s40644-025-00913-w (PMC12309162; doi:10.1186/s40644-025-00913-w)
Supplement: Supplementary file 4 — Supplementary Material 4 [file 40644_2025_913_MOESM4_ESM.docx]

**Supplementary Table 3*.* Comparison on the basis of LNM (n=412)**

| Variables | Total (n = 412) | Non-LNM  (n = 226) | LNM  (n = 186) | Statistics | *P* |
| --- | --- | --- | --- | --- | --- |
|  |  |  |  |  |  |
| Age, M (Q₁, Q₃) | 63.00 (57.00, 68.00) | 63.00 (58.00, 69.00) | 62.00 (56.00, 67.00) | Z=-1.73 | 0.084 |
| Sex, n(%) |  |  |  | χ²=0.50 | 0.479 |
| Male | 386 (93.69) | 210 (92.92) | 176 (94.62) |  |  |
| Female | 26 (6.31) | 16 (7.08) | 10 (5.38) |  |  |
| Smoking history, n(%) |  |  |  | χ²=7.17 | 0.028* |
| Never | 56 (13.59) | 38 (16.81) | 18 (9.68) |  |  |
| Ever | 104 (25.24) | 62 (27.43) | 42 (22.58) |  |  |
| Current | 252 (61.17) | 126 (55.75) | 126 (67.74) |  |  |
| Drinking history, n(%) |  |  |  | χ²=3.85 | 0.146 |
| Never | 140 (33.98) | 86 (38.05) | 54 (29.03) |  |  |
| Ever | 73 (17.72) | 39 (17.26) | 34 (18.28) |  |  |
| Current | 199 (48.30) | 101 (44.69) | 98 (52.69) |  |  |
| BMI, n(%) |  |  |  | χ²=18.22 | <0.001*** |
| Underweight | 29 (7.04) | 10 (4.42) | 19 (10.22) |  |  |
| Normal weight | 244 (59.22) | 121 (53.54) | 123 (66.13) |  |  |
| Overweight | 119 (28.88) | 80 (35.40) | 39 (20.97) |  |  |
| Obese | 20 (4.85) | 15 (6.64) | 5 (2.69) |  |  |
| NAT(Continuous), M (Q₁, Q₃) | 34.00 (20.84, 45.05) | 36.62(25.33, 47.25) | 31.45 (17.20, 42.38) | Z=-3.54 | <0.001*** |
| NAT, n(%) |  |  |  | χ²=15.68 | <0.001*** |
| Low NAT | 206 (50.00) | 93 (41.15) | 113 (60.75) |  |  |
| High NAT | 206 (50.00) | 133 (58.85) | 73 (39.25) |  |  |
| dNLR, M (Q₁, Q₃) | 1.56 (1.22, 2.10) | 1.50 (1.12, 2.01) | 1.76 (1.32, 2.34) | Z=-3.74 | <0.001*** |
| Tumor site, n(%) |  |  |  | χ²=52.75 | <0.001*** |
| HPSCC | 158 (38.35) | 51 (22.57) | 107 (57.53) |  |  |
| LSCC | 254 (61.65) | 175 (77.43) | 79 (42.47) |  |  |
| Z: Mann-Whitney test, χ²: Chi-square test, -: Fisher exact, M: Median, Q₁: 1st Quartile, Q₃: 3st Quartile, BMI body mass index, NAT neck adipose tissue, dNLR derived-Neutrophil to Lymphocyte Ratio; HPSCC LSCC  *P*<0.05 (*), *P*< 0.01(**), *P*< 0.001(***) | | | | | |
