# Supplementary material for: Inflammation as a mediator between neck adipose tissue and tumor aggressiveness in hypopharyngeal and laryngeal squamous cell carcinoma
Source: Cancer Imaging. 2025 Jul 29;25:95. doi: 10.1186/s40644-025-00913-w (PMC12309162; doi:10.1186/s40644-025-00913-w)
Supplement: Supplementary file 5 — Supplementary Material 5 [file 40644_2025_913_MOESM5_ESM.docx]

**Supplementary Table 4*.* Univariable and multivariable analyses for LNM (n=412)**

| Variables | Univariable analysis | | | | |  | Multivariable analysis | | | | |
| --- | --- | --- | --- | --- | --- | --- | --- | --- | --- | --- | --- |
|  | β | S.E | Z | P | OR (95%CI) |  | β | S.E | Z | P | Adjusted OR (95%CI) |
| BMI |  |  |  |  |  |  |  |  |  |  |  |
| Underweight |  |  |  |  | 1.00 (Reference) |  |  |  |  |  | 1.00 (Reference) |
| Normal weight | -0.63 | 0.41 | -1.52 | 0.128 | 0.54 (0.24 ~ 1.20) |  | -0.20 | 0.46 | -0.44 | 0.663 | 0.82 (0.33 ~ 2.03) |
| Overweight | -1.36 | 0.44 | -3.11 | 0.002** | 0.26 (0.11 ~ 0.60) |  | -0.68 | 0.52 | -1.32 | 0.188 | 0.51 (0.18 ~ 1.39) |
| Obesity | -1.74 | 0.65 | -2.69 | 0.007** | 0.18 (0.05 ~ 0.62) |  | -1.24 | 0.75 | -1.65 | 0.098 | 0.29 (0.07 ~ 1.26) |
| NAT |  |  |  |  |  |  |  |  |  |  |  |
| Low NAT |  |  |  |  | 1.00 (Reference) |  |  |  |  |  | 1.00 (Reference) |
| High NAT | -0.79 | 0.20 | -3.93 | <0.001*** | 0.45 (0.30 ~ 0.67) |  | -0.42 | 0.24 | -1.74 | 0.082 | 0.65 (0.41 ~ 1.06) |
| dNLR | 0.47 | 0.13 | 3.53 | <0.001*** | 1.60 (1.23 ~ 2.09) |  | 0.34 | 0.15 | 2.30 | 0.021* | 1.40 (1.05 ~ 1.86) |
| Dependent variable: LNM(lymph node metastasis), Adjusted covariates: sex, age, tumor site, smoking history, drinking history, BMI body mass index, NAT neck adipose tissue, dNLR derived-Neutrophil to Lymphocyte Ratio  OR: Odds Ratio, CI: Confidence Interval, *P*<0.05 (*), *P*< 0.01(**), *P*< 0.001(***) | | | | | | | | | | | |
