# Supplementary material for: Inflammation as a mediator between neck adipose tissue and tumor aggressiveness in hypopharyngeal and laryngeal squamous cell carcinoma
Source: Cancer Imaging. 2025 Jul 29;25:95. doi: 10.1186/s40644-025-00913-w (PMC12309162; doi:10.1186/s40644-025-00913-w)
Supplement: Supplementary file 6 — Supplementary Material 6 [file 40644_2025_913_MOESM6_ESM.docx]

**Supplementary Table 5**. **Comparison on the basis of tumor local invasion (n=412)**

| Variables | Total (n = 412) | Non-Invasion  (n = 238) | Invasion  (n = 174) | Statistics | *P* |
| --- | --- | --- | --- | --- | --- |
|  |  |  |  |  |  |
| Age, M (Q₁, Q₃) | 63.00 (57.00, 68.00) | 63.00 (57.25, 67.75) | 62.00 (56.00, 70.00) | Z=-0.11 | 0.912 |
| Sex, n(%) |  |  |  | χ²=1.49 | 0.221 |
| Male | 386 (93.69) | 220 (92.44) | 166 (95.40) |  |  |
| Female | 26 (6.31) | 18 (7.56) | 8 (4.60) |  |  |
| Smoking history, n(%) |  |  |  | χ²=1.35 | 0.509 |
| Never | 56 (13.59) | 36 (15.13) | 20 (11.49) |  |  |
| Ever | 104 (25.24) | 61 (25.63) | 43 (24.71) |  |  |
| Current | 252 (61.17) | 141 (59.24) | 111 (63.79) |  |  |
| Drinking history, n(%) |  |  |  | χ²=0.69 | 0.708 |
| Never | 140 (33.98) | 78 (32.77) | 62 (35.63) |  |  |
| Ever | 73 (17.72) | 45 (18.91) | 28 (16.09) |  |  |
| Current | 199 (48.30) | 115 (48.32) | 84 (48.28) |  |  |
| BMI, n(%) |  |  |  | χ²=22.34 | <0.001*** |
| Underweight | 29 (7.04) | 10 (4.20) | 19 (10.92) |  |  |
| Normal weight | 244 (59.22) | 128 (53.78) | 116 (66.67) |  |  |
| Overweight | 119 (28.88) | 83 (34.87) | 36 (20.69) |  |  |
| Obesity | 20 (4.85) | 17 (7.14) | 3 (1.72) |  |  |
| NAT(Continuous), M (Q₁, Q₃) | 34.00 (20.84, 45.05) | 36.75 (24.40, 47.28) | 31.07 (16.64, 42.11) | Z=-3.89 | <0.001*** |
| NAT, n(%) |  |  |  | χ²=19.26 | <0.001*** |
| Low NAT | 206 (50.00) | 97 (40.76) | 109 (62.64) |  |  |
| High NAT | 206 (50.00) | 141 (59.24) | 65 (37.36) |  |  |
| dNLR, M (Q₁, Q₃) | 1.56 (1.22, 2.10) | 1.46 (1.13, 1.89) | 1.89 (1.44, 2.50) | Z=-5.93 | <0.001*** |
| Tumor site, n(%) |  |  |  | χ²=0.58 | 0.444 |
| HPSCC | 158 (38.35) | 95 (39.92) | 63 (36.21) |  |  |
| LSCC | 254 (61.65) | 143 (60.08) | 111 (63.79) |  |  |
| Z: Mann-Whitney test, χ²: Chi-square test, -: Fisher exact, M: Median, Q₁: 1st Quartile, Q₃: 3st Quartile, BMI body mass index, NAT neck adipose tissue, dNLR derived-Neutrophil to Lymphocyte Ratio  *P*<0.05 (*), *P*< 0.01(**), *P*< 0.001(***) | | | | | |
