# Supplementary material for: Inflammation as a mediator between neck adipose tissue and tumor aggressiveness in hypopharyngeal and laryngeal squamous cell carcinoma
Source: Cancer Imaging. 2025 Jul 29;25:95. doi: 10.1186/s40644-025-00913-w (PMC12309162; doi:10.1186/s40644-025-00913-w)
Supplement: Supplementary file 7 — Supplementary Material 7 [file 40644_2025_913_MOESM7_ESM.docx]

**Supplementary Table 6.** **Univariable and multivariable analyses for tumor local invasion (n=412)**

| Variables | Univariable analysis | | | | |  | Adjusted multivariable analysis | | | | |
| --- | --- | --- | --- | --- | --- | --- | --- | --- | --- | --- | --- |
|  | β | S.E | Z | *P* | OR (95%CI) |  | β | S.E | Z | *P* | Adjusted OR (95%CI) |
| BMI |  |  |  |  |  |  |  |  |  |  |  |
| Underweight |  |  |  |  | 1.00 (Reference) |  |  |  |  |  | 1.00 (Reference) |
| Normal weight | -0.74 | 0.41 | -1.80 | 0.072 | 0.48 (0.21 ~ 1.07) |  | -0.53 | 0.45 | -1.17 | 0.242 | 0.59 (0.24 ~ 1.43) |
| Overweight | -1.48 | 0.44 | -3.37 | <0.001*** | 0.23 (0.10 ~ 0.54) |  | -1.03 | 0.51 | -2.04 | 0.042* | 0.36 (0.13 ~ 0.96) |
| Obesity | -2.38 | 0.74 | -3.22 | 0.001** | 0.09 (0.02 ~ 0.39) |  | -1.60 | 0.80 | -1.99 | 0.046* | 0.20 (0.04 ~ 0.97) |
| NAT |  |  |  |  |  |  |  |  |  |  |  |
| Low NAT |  |  |  |  | 1.00 (Reference) |  |  |  |  |  | 1.00 (Reference) |
| High  NAT | -0.89 | 0.20 | -4.35 | <0.001*** | 0.41 (0.27 ~ 0.61) |  | -0.64 | 0.24 | -2.65 | 0.008** | 0.53 (0.33 ~ 0.85) |
| dNLR | 0.85 | 0.15 | 5.77 | <0.001*** | 2.35 (1.76 ~ 3.13) |  | 0.83 | 0.16 | 5.27 | <0.001*** | 2.29 (1.68 ~ 3.12) |
| Dependent variable: tumor local invasion, Adjusted covariates: sex, age, tumor site, smoking history, drinking history; BMI body mass index, NAT neck adipose tissue, dNLR derived-Neutrophil to Lymphocyte Ratio  OR: Odds Ratio, CI: Confidence Interval, *P*<0.05 (*), *P*< 0.01(**), *P*< 0.001(***) | | | | | | | | | | | |
