# Supplementary material for: Inflammation as a mediator between neck adipose tissue and tumor aggressiveness in hypopharyngeal and laryngeal squamous cell carcinoma
Source: Cancer Imaging. 2025 Jul 29;25:95. doi: 10.1186/s40644-025-00913-w (PMC12309162; doi:10.1186/s40644-025-00913-w)
Supplement: Supplementary file 8 — Supplementary Material 8 [file 40644_2025_913_MOESM8_ESM.docx]

**Supplementary Table 7. Comparison of dNLR, BMI and NAT on the basis of TNM stage in male group (n=386)**

| Variables | Total (n = 386) | Early stage  (n = 105) | Advanced stage  (n = 281) | Statistic | *P* |
| --- | --- | --- | --- | --- | --- |
|  |  |  |  |  |  |
| dNLR, M (Q₁, Q₃) | 1.56 (1.23, 2.11) | 1.31 (1.04, 1.58) | 1.79 (1.34, 2.35) | Z=-6.16 | <0.001*** |
| BMI, n(%) |  |  |  | χ²=16.77 | <0.001*** |
| Underweight | 25 (6.48) | 0 (0.00) | 25 (8.90) |  |  |
| Normal weight | 229 (59.33) | 56 (53.33) | 173 (61.57) |  |  |
| Overweight | 113 (29.27) | 42 (40.00) | 71 (25.27) |  |  |
| Obesity | 19 (4.92) | 7 (6.67) | 12 (4.27) |  |  |
| NAT, n(%) |  |  |  | χ²=15.68 | <0.001*** |
| Low NAT | 193 (50.00) | 37 (35.24) | 156 (55.52) |  |  |
| High NAT | 193 (50.00) | 68 (64.76) | 125 (44.48) |  |  |
| Z: Mann-Whitney test, χ²: Chi-square test, M: Median, Q₁: 1st Quartile, Q₃: 3st Quartile, BMI body mass index, NAT neck adipose tissue, dNLR derived-Neutrophil to Lymphocyte Ratio, Invasion: Tumor local invasion  *P*<0.05 (*), *P*< 0.01(**), *P*< 0.001(***) | | | | | |
