# Supplementary material for: Inflammation as a mediator between neck adipose tissue and tumor aggressiveness in hypopharyngeal and laryngeal squamous cell carcinoma
Source: Cancer Imaging. 2025 Jul 29;25:95. doi: 10.1186/s40644-025-00913-w (PMC12309162; doi:10.1186/s40644-025-00913-w)
Supplement: Supplementary file 9 — Supplementary Material 9 [file 40644_2025_913_MOESM9_ESM.docx]

**Supplementary Table 8*.*** **Univariable and multivariable analyses for TNM stage in male group (n=386)**

| Variables | Univariable analysis | | | | |  | Multivariable analysis | | | | |  |  |
| --- | --- | --- | --- | --- | --- | --- | --- | --- | --- | --- | --- | --- | --- |
|  | β | S.E | Z | *P* | OR (95%CI) |  | β | S.E | Z | *P* | Adjusted OR (95%CI) |  |  |
| BMI |  |  |  |  |  |  |  |  |  |  |  |  |  |
| Underweight |  |  |  |  | 1.00 (Reference) |  |  |  |  |  |  |  |  |
| Normal weight | -16.44 | 791.24 | -0.02 | 0.983 | 0.00 (0.00 ~ Inf) |  |  |  |  |  |  |  |  |
| Overweight | -17.04 | 791.24 | -0.02 | 0.983 | 0.00 (0.00 ~ Inf) |  |  |  |  |  |  |  |  |
| Obese | -17.03 | 791.24 | -0.02 | 0.983 | 0.00 (0.00 ~ Inf) |  |  |  |  |  |  |  |  |
| NAT |  |  |  |  |  |  |  |  |  |  |  |  |  |
| Low NAT |  |  |  |  | 1.00 (Reference) |  |  |  |  |  | 1.00 (Reference) |  |  |
| High NAT | -0.83 | 0.24 | -3.50 | <0.001*** | 0.44 (0.27 ~ 0.69) |  | -0.59 | 0.26 | -2.26 | 0.024* | 0.56 (0.33 ~ 0.93) |  |  |
| dNLR | 1.24 | 0.23 | 5.49 | <0.001*** | 3.44 (2.21 ~ 5.35) |  | 1.15 | 0.23 | 4.93 | <0.001*** | 3.15 (2.00 ~ 4.97) |  |  |
| Dependent variable: TNM stage (male). Adjusted covariates: age, tumor site, smoking history, drinking history, BMI body mass index, NAT neck adipose tissue, dNLR derived-Neutrophil to Lymphocyte Ratio  OR: Odds Ratio, CI: Confidence Interval, *P*<0.05 (*), *P*< 0.01(**), *P*< 0.001(***) | | | | | | | | | | | |  |  |
