# Supplementary material for: Inflammation as a mediator between neck adipose tissue and tumor aggressiveness in hypopharyngeal and laryngeal squamous cell carcinoma
Source: Cancer Imaging. 2025 Jul 29;25:95. doi: 10.1186/s40644-025-00913-w (PMC12309162; doi:10.1186/s40644-025-00913-w)
Supplement: Supplementary file 10 — Supplementary Material 10 [file 40644_2025_913_MOESM10_ESM.docx]

**Supplementary Table 9*.* Comparison of dNLR, BMI and NAT on the basis of LNM in male group (n=386)**

| Variables | Total (n = 386) | non-LNM  (n = 210) | LNM  (n = 176) | Statistic | *P* |
| --- | --- | --- | --- | --- | --- |
|  |  |  |  |  |  |
| dNLR, M (Q₁, Q₃) | 1.56 (1.23, 2.11) | 1.50 (1.14, 1.99) | 1.83 (1.34, 2.36) | Z=-3.97 | <0.001*** |
| BMI, n(%) |  |  |  | χ²=21.26 | <0.001*** |
| Underweight | 25 (6.48) | 7 (3.33) | 18 (10.23) |  |  |
| Normal weight | 229 (59.33) | 112 (53.33) | 117 (66.48) |  |  |
| Overweight | 113 (29.27) | 77 (36.67) | 36 (20.45) |  |  |
| Obese | 19 (4.92) | 14 (6.67) | 5 (2.84) |  |  |
| NAT, n(%) |  |  |  | χ²=18.42 | <0.001*** |
| Low NAT | 193 (50.00) | 84 (40.00) | 109 (61.93) |  |  |
| High NAT | 193 (50.00) | 126 (60.00) | 67 (38.07) |  |  |
| Z: Mann-Whitney test, χ²: Chi-square test, M: Median, Q₁: 1st Quartile, Q₃: 3st Quartile, LNM lymph node metastasis, BMI body mass index, NAT neck adipose tissue, dNLR derived-Neutrophil to Lymphocyte Ratio  *P*<0.05 (*), *P*< 0.01(**), *P*< 0.001(***) | | | | | |
