# Supplementary material for: Inflammation as a mediator between neck adipose tissue and tumor aggressiveness in hypopharyngeal and laryngeal squamous cell carcinoma
Source: Cancer Imaging. 2025 Jul 29;25:95. doi: 10.1186/s40644-025-00913-w (PMC12309162; doi:10.1186/s40644-025-00913-w)
Supplement: Supplementary file 11 — Supplementary Material 11 [file 40644_2025_913_MOESM11_ESM.docx]

**Supplementary Table 10*.* Univariable and multivariable analyses for LNM in male group (n=386)**

| Variables | Univariable analysis | | | | |  | Multivariable analysis | | | | |
| --- | --- | --- | --- | --- | --- | --- | --- | --- | --- | --- | --- |
|  | β | S.E | Z | *P* | OR (95%CI) |  | β | S.E | Z | *P* | Adjusted OR (95%CI) |
| BMI |  |  |  |  |  |  |  |  |  |  |  |
| Underweight |  |  |  |  | 1.00 (Reference) |  |  |  |  |  | 1.00 (Reference) |
| Normal weight | -0.90 | 0.46 | -1.94 | 0.053 | 0.41 (0.16 ~ 1.01) |  | -0.43 | 0.52 | -0.82 | 0.410 | 0.65 (0.24 ~ 1.80) |
| Overweight | -1.70 | 0.49 | -3.49 | <0.001^***^ | 0.18 (0.07 ~ 0.47) |  | -0.95 | 0.57 | -1.67 | 0.095 | 0.39 (0.13 ~ 1.18) |
| Obese | -1.97 | 0.69 | -2.88 | 0.004^**^ | 0.14 (0.04 ~ 0.53) |  | -1.26 | 0.80 | -1.58 | 0.114 | 0.28 (0.06 ~ 1.35) |
| NAT |  |  |  |  |  |  |  |  |  |  |  |
| Low NAT |  |  |  |  | 1.00 (Reference) |  |  |  |  |  | 1.00 (Reference) |
| High NAT | -0.89 | 0.21 | -4.26 | <0.001^***^ | 0.41 (0.27 ~ 0.62) |  | -0.51 | 0.25 | -1.99 | 0.046^*^ | 0.60 (0.37 ~ 0.99) |
| dNLR | 0.52 | 0.14 | 3.71 | <0.001^***^ | 1.68 (1.28 ~ 2.21) |  | 0.38 | 0.15 | 2.49 | 0.013^**^ | 1.46 (1.09 ~ 1.98) |
| Dependent variable: LNM (male, lymph node metastasis). Adjusted covariates: age, tumor site, smoking history, drinking history, BMI body mass index, NAT neck adipose tissue, dNLR derived-Neutrophil to Lymphocyte Ratio  OR: Odds Ratio, CI: Confidence Interval, *P*<0.05 (*), *P*< 0.01(**), *P*< 0.001(***) | | | | | | | | | | | |
