# Supplementary material for: Inflammation as a mediator between neck adipose tissue and tumor aggressiveness in hypopharyngeal and laryngeal squamous cell carcinoma
Source: Cancer Imaging. 2025 Jul 29;25:95. doi: 10.1186/s40644-025-00913-w (PMC12309162; doi:10.1186/s40644-025-00913-w)
Supplement: Supplementary file 12 — Supplementary Material 12 [file 40644_2025_913_MOESM12_ESM.docx]

**Supplementary Table 11*.* Comparison of dNLR, BMI and NAT on the basis of tumor local invasion in male group (n=386)**

| Variables | Total (n = 386) | non-invasion  (n = 220) | invasion  (n = 166) | Statistic | *P* |
| --- | --- | --- | --- | --- | --- |
|  |  |  |  |  |  |
| dNLR, M (Q₁, Q₃) | 1.56 (1.23, 2.11) | 1.46 (1.15, 1.91) | 1.87 (1.44, 2.50) | Z=-5.45 | <0.001*** |
| BMI, n(%) |  |  |  | χ²=21.68 | <0.001*** |
| Underweight | 25 (6.48) | 9 (4.09) | 16 (9.64) |  |  |
| Normal weight | 229 (59.33) | 116 (52.73) | 113 (68.07) |  |  |
| Overweight | 113 (29.27) | 79 (35.91) | 34 (20.48) |  |  |
| Obese | 19 (4.92) | 16 (7.27) | 3 (1.81) |  |  |
| NAT, n(%) |  |  |  | χ²=15.26 | <0.001*** |
| Low NAT | 193 (50.00) | 91 (41.36) | 102 (61.45) |  |  |
| High NAT | 193 (50.00) | 129 (58.64) | 64 (38.55) |  |  |
| Z: Mann-Whitney test, χ²: Chi-square test, M: Median, Q₁: 1st Quartile, Q₃: 3st Quartile, Invasion tumor local invasion, BMI body mass index, NAT neck adipose tissue, dNLR derived-Neutrophil to Lymphocyte Ratio  *P*<0.05 (*), *P*< 0.01(**), *P*< 0.001(***) | | | | | |
