# Supplementary material for: Inflammation as a mediator between neck adipose tissue and tumor aggressiveness in hypopharyngeal and laryngeal squamous cell carcinoma
Source: Cancer Imaging. 2025 Jul 29;25:95. doi: 10.1186/s40644-025-00913-w (PMC12309162; doi:10.1186/s40644-025-00913-w)
Supplement: Supplementary file 13 — Supplementary Material 13 [file 40644_2025_913_MOESM13_ESM.docx]

**Supplementary Table 12*.* Univariable and multivariable analyses for tumor local invasion in male group (n=386)**

| Variables | Univariable analysis | | | | |  | Multivariable analysis | | | | |
| --- | --- | --- | --- | --- | --- | --- | --- | --- | --- | --- | --- |
|  | β | S.E | Z | *P* | OR (95%CI) |  | β | S.E | Z | *P* | Adjusted OR (95%CI) |
| BMI |  |  |  |  |  |  |  |  |  |  |  |
| Underweight |  |  |  |  | 1.00 (Reference) |  |  |  |  |  | 1.00 (Reference) |
| Normal weight | -0.60 | 0.44 | -1.38 | 0.169 | 0.55 (0.23 ~ 1.29) |  | -0.35 | 0.47 | -0.74 | 0.462 | 0.71 (0.28 ~ 1.79) |
| Overweight | -1.42 | 0.46 | -3.05 | 0.002** | 0.24 (0.10 ~ 0.60) |  | -0.96 | 0.53 | -1.82 | 0.068 | 0.38 (0.14 ~ 1.08) |
| Obese | -2.25 | 0.75 | -2.98 | 0.003** | 0.11 (0.02 ~ 0.46) |  | -1.47 | 0.82 | -1.80 | 0.071 | 0.23 (0.05 ~ 1.14) |
| NAT |  |  |  |  |  |  |  |  |  |  |  |
| Low NAT |  |  |  |  | 1.00 (Reference) |  |  |  |  |  | 1.00 (Reference) |
| High NAT | -0.82 | 0.21 | -3.88 | <0.001*** | 0.44 (0.29 ~ 0.67) |  | -0.52 | 0.25 | -2.12 | 0.034** | 0.59 (0.37 ~ 0.96) |
| dNLR | 0.80 | 0.15 | 5.32 | <0.001*** | 2.22 (1.65 ~ 2.98) |  | 0.77 | 0.16 | 4.82 | <0.001*** | 2.16 (1.58 ~ 2.95) |
| Dependent variable: tumor local invasion (male). Adjusted covariates: age, tumor site, smoking history, drinking history, BMI body mass index, NAT neck adipose tissue, dNLR derived-Neutrophil to Lymphocyte Ratio  OR: Odds Ratio, CI: Confidence Interval, *P*<0.05 (*), *P*< 0.01(**), *P*< 0.001(***) | | | | | | | | | | | |
