# Supplementary material for: Inflammation as a mediator between neck adipose tissue and tumor aggressiveness in hypopharyngeal and laryngeal squamous cell carcinoma
Source: Cancer Imaging. 2025 Jul 29;25:95. doi: 10.1186/s40644-025-00913-w (PMC12309162; doi:10.1186/s40644-025-00913-w)
Supplement: Supplementary file 14 — Supplementary Material 14 [file 40644_2025_913_MOESM14_ESM.docx]

**Supplementary Figure 1*.* Evaluation of LNM based on the enhanced CT images.**

Evaluation of LNM based on the enhanced CT images (white arrows). A. The short diameter of LN larger than or equal to 10 mm. B. LN with Central necrosis or a contrast-enhancing rim. C. LNs with indistinct nodal margins and infiltration into local tissue. LN lyphm node; LNM lymph node metastasis.

**Supplementary Figure 2*.* Evaluation of tumor local invasion based on the enhanced CT image.**

Evaluation of tumor local invasion based on the enhanced CT image. A. Right thyroid cartilage with erosion and lysis. B. Cricoid cartilage with asymmetric sclerosis. C. Right arytenoid cartilage with asymmetric sclerosis and right thyroid cartilage was spread through by the tumor. D. Extralaryngeal tumor spread: the primary tumor extended into right thyroid gland.

**Supplementary Figure 3*.* Comparison of dNLR, BMI and NAT on the basis of TNM stage, LNM and tumor local invasion in male group.**

Comparison of BMI(A), NAT(B) and dNLR(C) on the basis of TNM stage, LNM and tumor local invasion in male demonstrated significant differences (n=386). BMI body mass index, NAT neck adipose tissue, dNLR derived-Neutrophil to Lymphocyte Ratio, LNM lymph node metastasis, Invasion, tumor local invasion. *P* < 0.05 (*), *P* < 0.01(**), *P* < 0.001(***)
