# Supplementary figures and images for: Inflammation as a mediator between neck adipose tissue and tumor aggressiveness in hypopharyngeal and laryngeal squamous cell carcinoma
Source: Cancer Imaging. 2025 Jul 29;25:95. doi: 10.1186/s40644-025-00913-w (PMC12309162; doi:10.1186/s40644-025-00913-w)

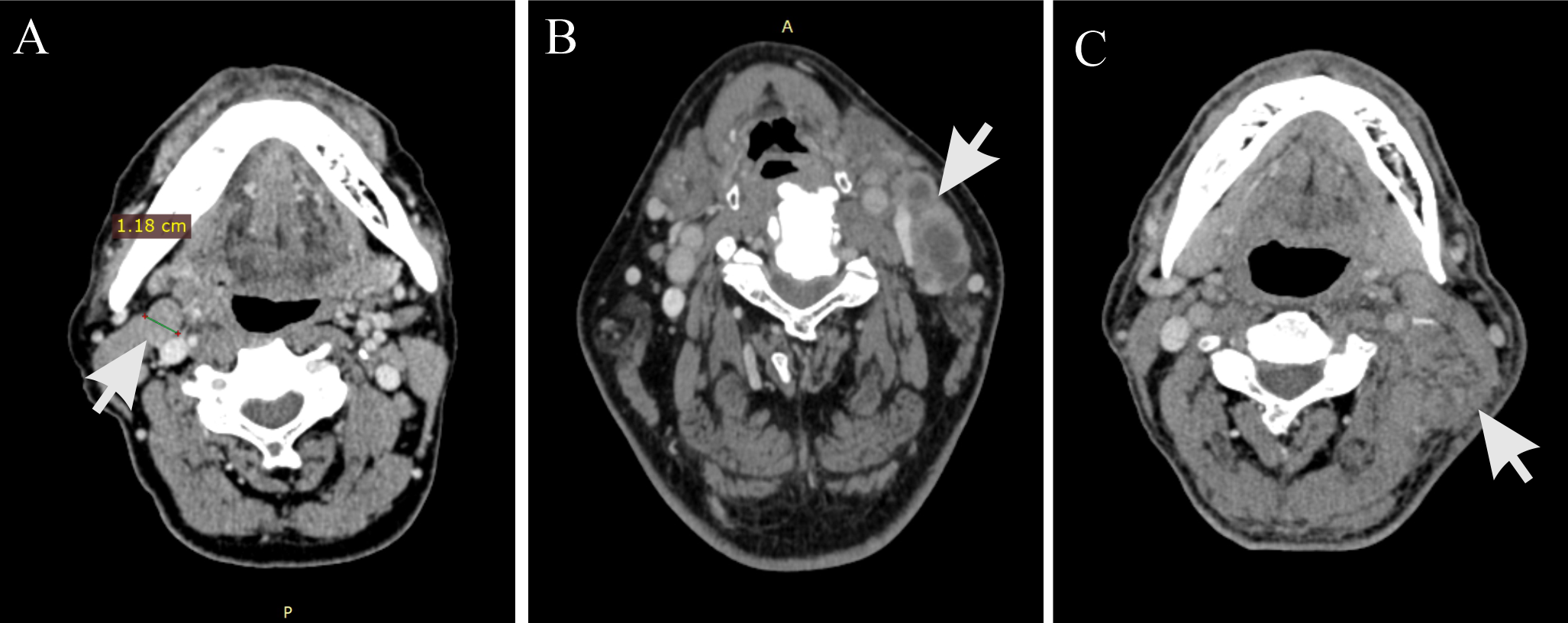

Supplement: Supplementary file 15 — Supplementary Material 15 [file 40644_2025_913_MOESM15_ESM.tif]

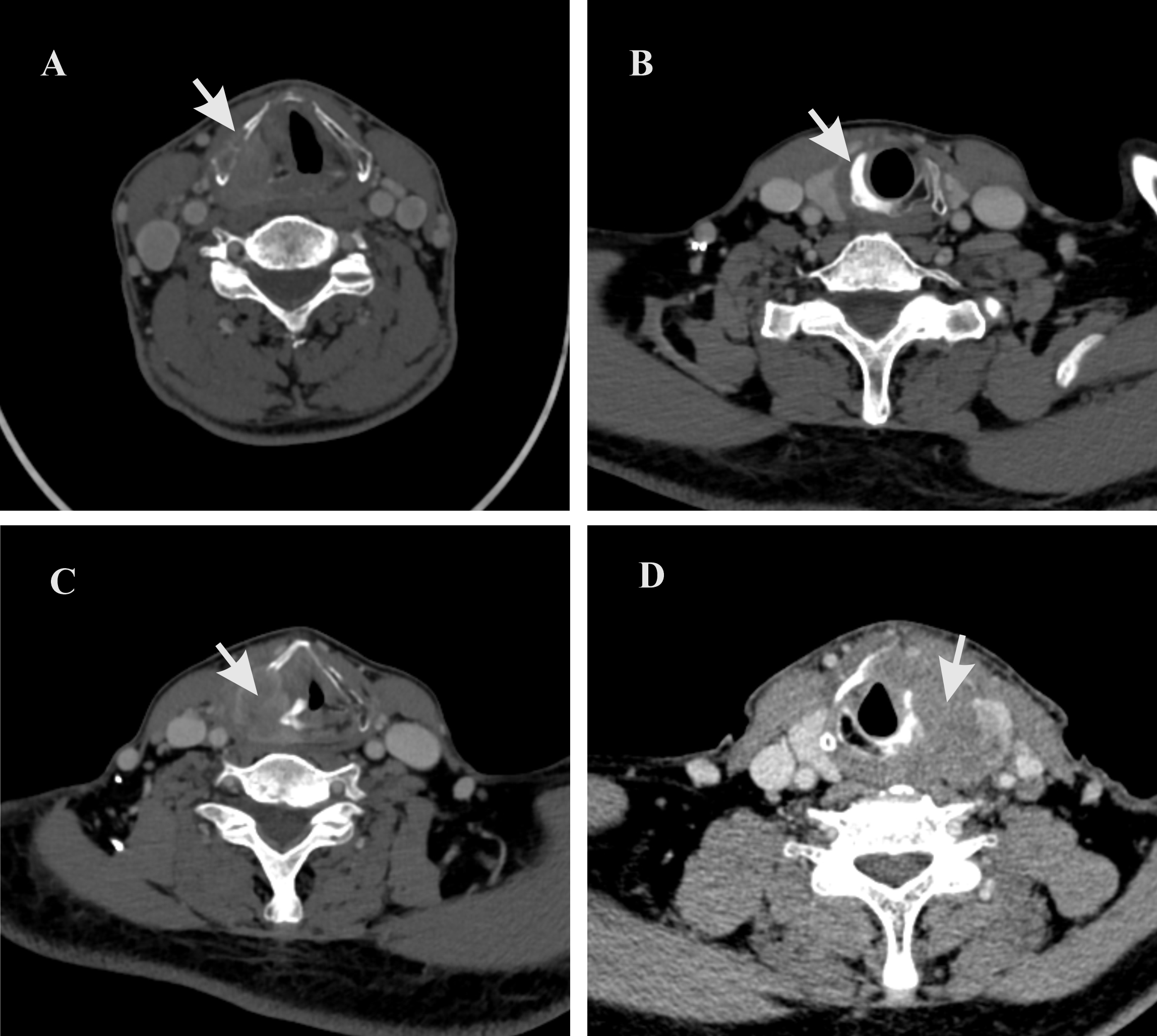

Supplement: Supplementary file 16 — Supplementary Material 16 [file 40644_2025_913_MOESM16_ESM.tif]

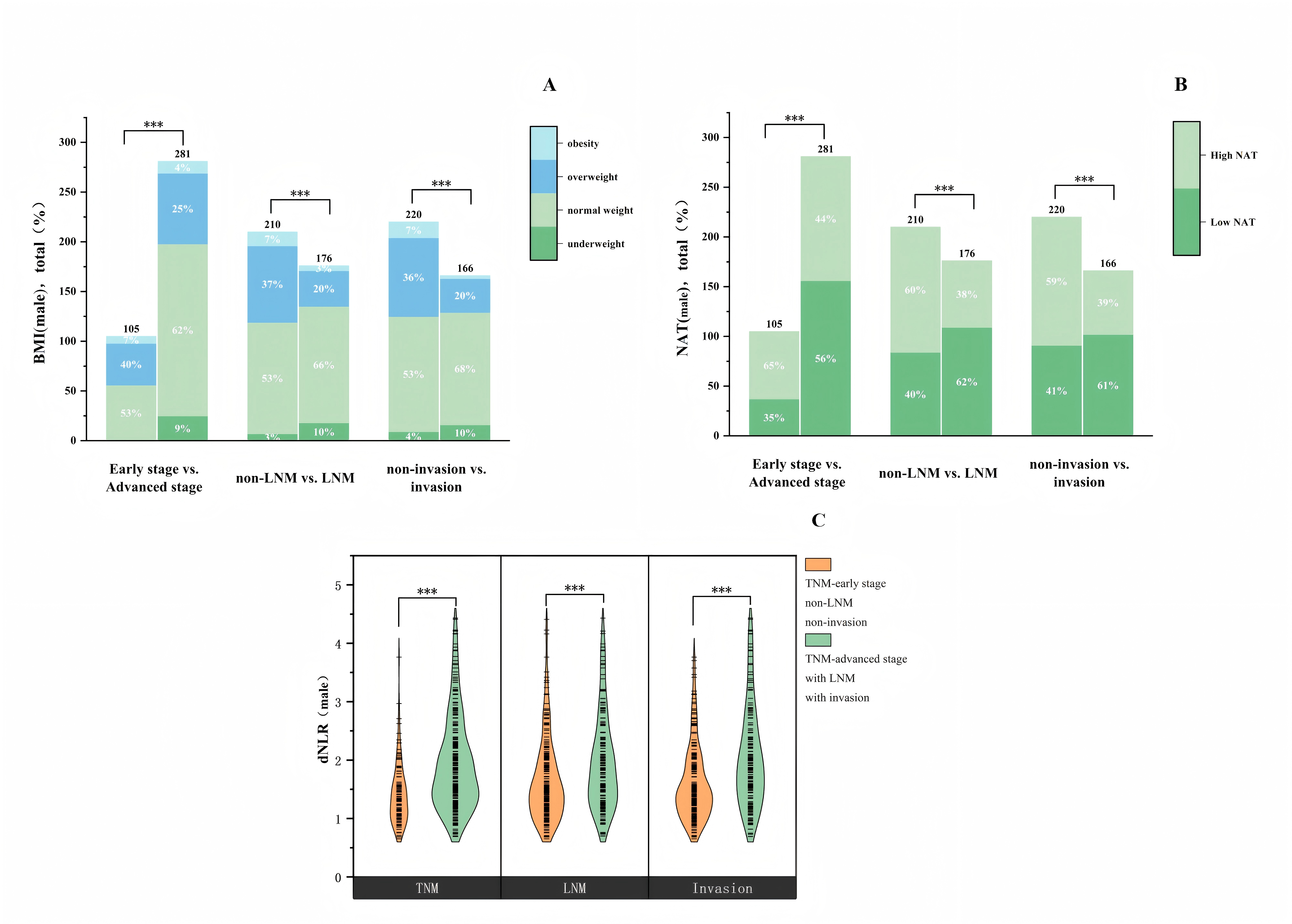

Supplement: Supplementary file 17 — Supplementary Material 17 [file 40644_2025_913_MOESM17_ESM.jpg]
